# Bariatric Surgery Improves the Atherogenic Profile of Circulating Methylarginines in Obese Patients: Results from a Pilot Study

**DOI:** 10.3390/metabo11110759

**Published:** 2021-11-04

**Authors:** Julie Poirier, Chloé Cloteau, Audrey Aguesse, Xavier Billot, Etienne Thévenot, Michel Krempf, René Valéro, Marie Maraninchi, Mikaël Croyal

**Affiliations:** 1 CRNH-Ouest Mass Spectrometry Core Facility, F-44000 Nantes, France; jpoirier4935@gmail.com (J.P.); chloe.cloteau@oniris-nantes.fr (C.C.); audrey.aguesse@gmail.com (A.A.); xbillot@gmail.com (X.B.); Michel.KREMPF@clinique-breteche.fr (M.K.); 2 CEA, LIST, Laboratory for Data Sciences and Decision, MetaboHUB-Paris, F-91190 Gif-sur-Yvette, France; Etienne.THEVENOT@cea.fr; 3 ELSAN, Clinique Bretéché, F-44000 Nantes, France; 4 Aix Marseille Univ, APHM, INSERM, INRAE, C2VN, Department of Nutrition, Metabolic Diseases and Endocrinology, University Hospital La Conception, F-13385 Marseille, France; Rene.VALERO@ap-hm.fr; 5 CNRS, INSERM, l’Institut du Thorax, Nantes University, F-44000 Nantes, France; 6 CHU Nantes, Inserm, CNRS, SFR Santé, Nantes University, Inserm UMS 016, CNRS UMS 3556, F-44000 Nantes, France

**Keywords:** bariatric surgery, ADMA, SDMA, NMMA, DMGV, methylarginines

## Abstract

Bariatric surgery improves obesity-related comorbidities. Methylarginines are biomarkers of cardiometabolic risk, liver steatosis, and insulin resistance. Here, we aimed to investigate methylarginines in obese patients undergoing bariatric surgery and compared them to age- and sex-matched healthy subjects. Thirty-one obese patients who underwent bariatric surgery and 31 healthy individuals were used for this retrospective study. The basal serum methylarginine levels were determined in the healthy individuals and the obese patients, before surgery and 6 and 12 months after surgery, by mass spectrometry. Compared with the healthy individuals, the obese patients displayed elevated monomethylarginine (mean change: +95%, *p* < 0.001), asymmetric-dimethylarginine (+105%, *p* < 0.001), symmetric-dimethylarginine (+25%, *p* = 0.003), and dimethylguanidino valerate (+32%, *p* = 0.008) concentrations. Bariatric surgery durably reduced the body mass index by 28% (12 months, 95%CI: 24–33, *p* = 0.002) and improved plasma lipids, insulin resistance, and liver function. Bariatric surgery reduced the serum levels of monomethylarginine and asymmetric-dimethylarginine by 12% (95%CI: 6–17) and 36% (95%CI: 27–45) (12 months, *p* = 0.003), respectively, but not symmetric-dimethylarginine or dimethylguanidino valerate. The monomethylarginine and asymmetric-dimethylarginine concentrations were strongly correlated with markers of dyslipidemia, insulin resistance, and a fatty liver. Serum dimethylguanidino valerate was primarily correlated with glycemia and renal function, whereas serum symmetric-dimethylarginine was almost exclusively associated with renal function. In conclusion, the monomethylarginine and asymmetric-dimethylarginine levels are efficiently decreased by bariatric surgery, leading to a reduced atherogenic profile in obese patients. Methylarginines follow different metabolic patterns, which could help for the stratification of cardiometabolic disorders in obese patients.

## 1. Introduction

Obesity is frequently associated with metabolic complications such as type 2 diabetes (T2D), hypertension, and dyslipidemia, leading to a reduction in life expectancy [1]. In addition, the prevalence of nonalcoholic fatty liver disease (NAFLD) tends to increase with that of obesity, dyslipidemia, and diabetes [2]. Regardless of the risk of developing cirrhosis and/or liver cancer, NAFLD has also been reported as an independent risk factor for atherosclerosis [3,4,5]. Altogether, these complications overexpose obese patients to an increased risk of cardiovascular disease (CVD), but the interactions between NAFLD and CVD remain poorly understood [6]. The detection of the early biomarkers of these diseases could help to better understand the underlying mechanisms and lead to better care for the most at-risk patients [7].

Bariatric surgery is considered the most effective treatment for severely obese patients with a body mass index (BMI) of ≥40 kg/m^2^ or ≥35 kg/m^2^ associated with at least one comorbidity that can be improved by surgery; in severely obese patients for whom well-conducted medical, nutritional, dietetic, and psychotherapeutic treatments failed for 6–12 months; or in severely obese patients displaying an absence of sufficient weight loss or in the absence of the maintenance of weight loss [8,9,10,11]. Several surgical methods have been developed, but sleeve gastrectomy (SG) and gastric bypass (GBP) are the two most commonly used procedures in clinical practice, even if the choice of the surgery is still debated [8,9]. SG and GBP lead to a significant BMI reduction and improvements in associated comorbidities such as high blood pressure, diabetes, and dyslipidemia [12]. Both SG and GBP decrease plasma triglyceride (TG) levels and increase high-density lipoprotein cholesterol (HDL-C) levels, but the decrease in low-density lipoprotein cholesterol (LDL-C) levels is often more pronounced after a malabsorptive procedure (e.g., GBP) [12].

With the development of metabolomics, numerous biomarkers of metabolic dysfunction and CVD have been discovered. Methylarginines, especially asymmetric dimethylarginine (ADMA), have been shown to predict CVD risk [13]. Methylarginines are produced by the degradation of methylated proteins originating from post-translational modifications [14]. These reactions lead to the formation of monomethylarginine (NMMA), ADMA, and symmetric dimethylarginine (SDMA), and elevated serum concentrations of ADMA and SDMA have been reported to be associated with CVD and renal complications, respectively [13,15]. ADMA can be metabolized to citrulline (CIT) and dimethylguanidino valerate (DMGV) [14], which has recently been identified as an independent biomarker of NAFLD [7]. In a cohort of individuals undergoing GBP surgery, the circulating DMGV level was found to be correlated with nonalcoholic steatohepatitis, and it significantly decreased in parallel with the improvement of cardiometabolic risk. The baseline DMGV plasma concentration also independently predicted new-onset diabetes within the next 12 years in these patients [7]. However, whether the modulation of the plasma DMGV concentration could stem from severe liver damage or could be an independent biomarker of obesity-related metabolic complications remains an open question.

Here we studied the modulation of the methylarginine concentrations in a varied population of morbidly obese patients undergoing GBP or SG surgery. The concentrations of the main methylarginine metabolites were investigated before and after surgery and compared with those of age- and sex-matched healthy individuals.

## 2. Results

### 2.1. Obesity Increased the Serum Concentrations of Methylarginines

The anthropometric and biochemical parameters of the subjects are shown in Table 1. The healthy and obese subjects were similar with respect to sex ratio and age, but they were significantly different with respect to BMI (*p* = 0.003). Compared with the healthy individuals, the obese patients displayed a disturbed lipid profile with an increase in TG (+32%, *p* = 0.003) and LDL-C (+26%, *p* = 0.003), and a decrease in HDL-C (−44%, *p* = 0.003). In addition, the transaminase serum concentrations (aspartate aminotransferase (ASAT), +36%, *p* = 0.027; alanine aminotransferase (ALAT), +27%, *p* = 0.005) as well as the homeostasis model assessment of insulin resistance (HOMA-IR, +69%, *p* = 0.006) were significantly increased in the obese patients. Furthermore, an analysis of the NAFLD fibrosis scores (NAFLD FS) in the obese individuals revealed that five patients (16%, including two patients with type 2 diabetes (T2D)) displayed advanced liver fibrosis, while seven of them (23%, including one patient with T2D) did not present any signs of liver disease. In line with traditional guidelines, we assumed that the remaining individuals (61%, including six patients with T2D) had intermediate levels of liver fibrosis.

**Figure 1:**
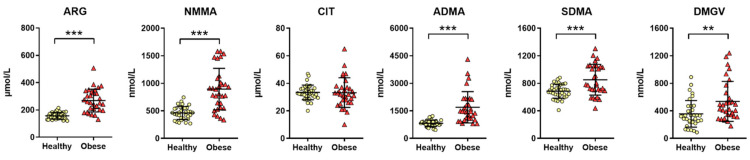
Comparison of the methylarginine metabolite plasma concentrations between the healthy and obese subjects at baseline. **: *p* < 0.01; ***: *p* < 0.001 (Mann–Whitney test, Benjamini–Hochberg correction for multiple testing). ARG, arginine; NMMA, N-monomethylarginine; CIT, citrulline; ADMA, asymmetric dimethylarginine; SDMA, symmetric dimethylarginine; DMGV, dimethylguanidino valerate.

As shown in Figure 1, the serum concentrations of methylated arginine metabolites were significantly increased in the obese patients compared with the healthy individuals, with the exception of CIT. Compared with the healthy individuals, the serum concentrations of arginine (ARG), NMMA, ADMA, SDMA, and DMGV were significantly increased by 70% (*p* < 0.001), 95% (*p* < 0.001), 105% (*p* < 0.001), 25% (*p* = 0.003), and 32% (*p* = 0.006), respectively, in the obese patients. The serum levels of methylarginines were not significantly different according to the diabetes status of the obese patients, except for DMGV which was higher in patients with T2D (median (range): 663 (266–1239) nmol/L (*n* = 9) vs. 398 (180–1188) nmol/L (*n* = 22), *p* = 0.044). The serum levels of methylarginines were not significantly different in obese patients with advanced liver fibrosis (*n* = 5).

### 2.2. Bariatric Surgery Improved the Atherogenic Profile of Methylarginines

As expected and shown in Table 2, bariatric surgery strongly reduced and stabilized the BMI by 24% to 29% (*p* = 0.002) over 12 months. In addition, bariatric surgery improved the lipid profiles by reducing the TC by 11% (*p* = 0.002), TG by 36% (*p* = 0.004), and LDL-C by 19% (*p* = 0.008), and by increasing HDL-C by 31% (*p* = 0.027). Bariatric surgery also decreased the HOMA-IR by 71% (*p* = 0.003) and improved liver function by decreasing the transaminase serum concentrations of ASAT and ALAT by 15% (*p* = 0.031) and 31% (*p* = 0.004), respectively. Moreover, the NAFLD FS was decreased by 92% (*p* = 0.002). Bariatric surgery also improved the renal function by increasing the estimated glomerular filtration rate (eGFR) by 6% (*p* = 0.004). Of note, we did not find any significant difference between SG and GBP, except for the reduction in LDL-C, and TC concentrations (*p* < 0.01), which were only significant with GBP (−28% and −18% over 12 months, respectively; *p* = 0.002).

**Figure 2:**
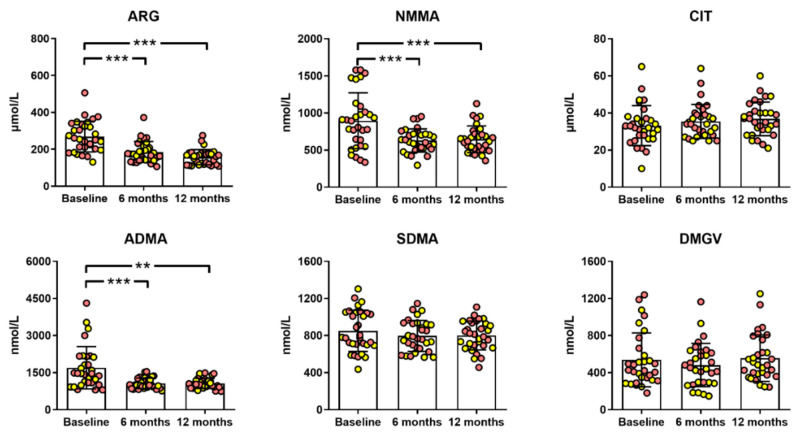
Benefits of bariatric surgery on serum methylarginines. **: *p* < 0.01; ***: *p* < 0.001 (Friedman test, Benjamini–Hochberg correction for multiple testing). ARG, arginine; NMMA, N-monomethylarginine; CIT, citrulline; ADMA, asymmetric dimethylarginine; SDMA, symmetric dimethylarginine; DMGV, dimethylguanidino valerate. Yellow and red dots indicate patients subjected to a sleeve gastrectomy or a gastric bypass, respectively.

Independent of the surgical procedure, bariatric surgery reduced the serum concentrations of atherogenic ADMA and related metabolites (ARG and NMMA) but not CIT, SDMA, or DMGV (Figure 2). After surgery, the serum concentrations of ARG, NMMA, and ADMA were significantly reduced by 36% (*p* = 0.002), 30% (*p* = 0.002), and 30% (*p* = 0.002), respectively. The serum concentrations of NMMA and ADMA remained higher in the obese patients after bariatric surgery compared to the healthy individuals (Table 3), in line with their BMI values, which were still greater than 30 kg/m^2^. Of note, no significant differences were found between patients with or without T2D, nor between patients with or without advanced liver fibrosis.

### 2.3. Correlations of Methylarginine Concentrations with Clinical Data

**Figure 3:**
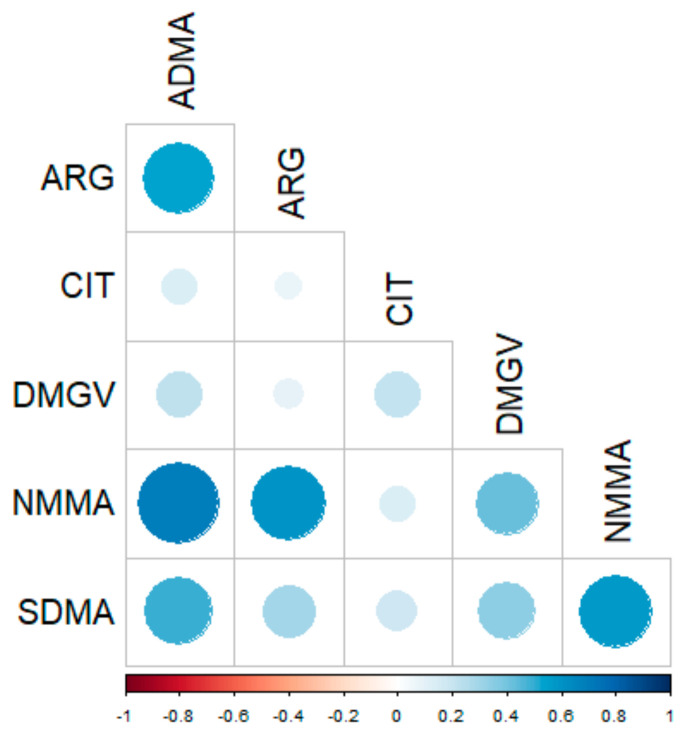
Spearman correlations obtained between methylarginine metabolites (all values, *n* = 122). ARG, arginine; NNMA, N-mono-methylarginine; CIT, citrulline; ADMA, asymmetric dimethylarginine; SDMA, symmetric dimethylarginine; DMGV, dimethylguanidino valerate.

The multiple Spearman correlations (all values including healthy and obese patients, before/after surgery, *n* = 122) underlined the interconnections between all target metabolites (Figure 3) and confirmed that the serum concentration of ADMA is significantly correlated with its precursors (i.e., ARG, r = 0.56, *p* < 0.001 and NMMA, r = 0.67, *p* < 0.001) and with its main metabolite, DMGV (r = 0.23; *p* = 0.011). Of note, these correlations remained significant in healthy individuals and in obese patients analyzed separately.

**Figure 4:**
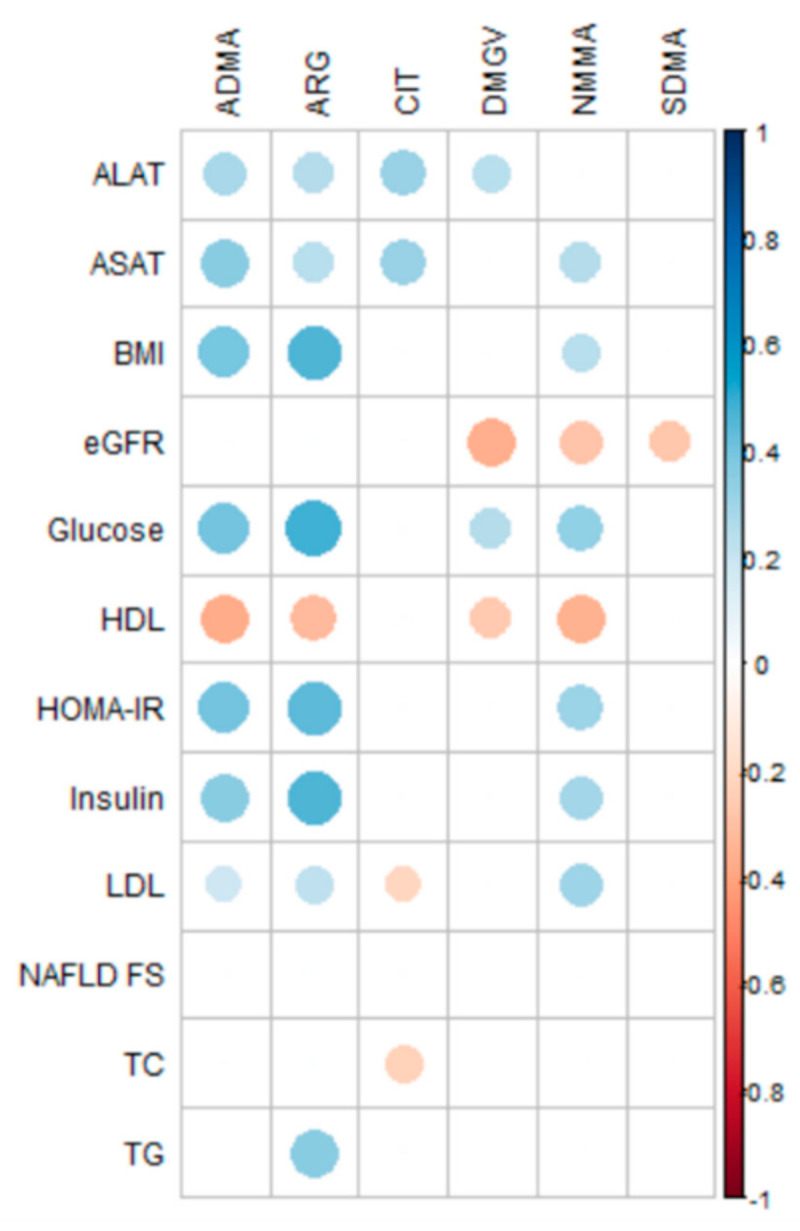
Spearman correlations obtained between the methylarginine metabolites and the main clinical parameters of the obese patients (all values, *n* = 122). Blue and red colors indicate positive and negative correlations, respectively. Only significant correlations are presented (*p* < 0.05). The size of the circles indicates significance. NMMA, N-monomethylarginine; ADMA, asymmetric dimethylarginine; SDMA, symmetric dimethylarginine; DMGV, dimethylguanidino valerate; BMI: body mass index; TC: total cholesterol; TG: triglycerides; HDL-C: high-density lipoprotein cholesterol; LDL-C: low-density lipoprotein cholesterol; ASAT: aspartate aminotransferase; ALAT: alanine aminotransferase; HOMA-IR, homeostasis model assessment of insulin resistance, NAFLD FC, nonalcoholic fatty liver disease fibrosis score.

As shown in Figure 4, ARG, NMMA, and ADMA were similarly correlated with BMI (r = 0.25–0.46; *p* < 0.022), insulin resistance (r = 0.31–0.44; *p* < 0.01), liver parameters (r = 0.25–0.35; *p* < 0.021), and plasma lipids, especially LDL-C (r = 0.20–0.31; *p* < 0.05) and HDL-C (r = −0.33 to −0.38; *p* < 0.001). Meanwhile, DMGV was primarily correlated with glycemia (r = 0.26; *p* = 0.036) and HDL-C (r = −0.27; *p* = 0.003). In sharp contrast, SDMA was correlated with the eGFR (r = −0.27; *p* = 0.010) but not with plasma lipids. Of note, NMMA and DMGV were also negatively correlated with the eGFR in this study (r = −0.29 and r = −0.37, respectively; *p* < 0.006). However, by considering the absolute changes brought by bariatric surgery (6 and 12 months), only the changes in the ARG and NMMA serum levels were significantly associated with those of the HOMA-IR (r = 0.46, *p* = 0.002 and r = 0.32, *p* = 0.043; respectively).

## 3. Discussion

Methylarginines are promising biomarkers of metabolic disorders leading to cardiometabolic burden. Compared to the healthy individuals, we showed that the obese patients displayed elevated serum levels of ARG, NMMA, ADMA, SDMA, and DMGV, as well as traditional markers of dyslipidemia, insulin resistance, and a fatty liver. In this retrospective study, we confirmed that bariatric surgery greatly improved the metabolic profiles of obese patients and led to a sharp reduction in the serum concentrations of ARG, NMMA, and ADMA in parallel with their BMI, regardless of the surgical method. The serum concentrations of ARG, NMMA, and ADMA were found to be correlated with a wide range of clinical markers of metabolic disorders. In contrast, the serum levels of DMGV were mainly correlated with glycemia and renal function, whereas the serum levels of SDMA were almost exclusively associated with renal function. However, this study was not powered or designed to evaluate the probable associations between the circulating metabolites and the diets, comorbidities, or medications of the patients, even if they would determine broader insights into the physiology of bariatric surgery. Besides, the healthy individuals were recruited a posteriori, which forces us to be cautious about the result interpretations. In this respect, the current study must be considered a retrospective and observational pilot study. Further stratification analyses by different surgeries, gender, age range, comorbidities, and obesity severity will be necessary for larger cohorts to provide broader insights into the physiology of bariatric surgery.

NMMA, SDMA, and ADMA originate from post-translational methylations of ARG residues within proteins, which are catalyzed by protein arginine methyltransferases (PRMTs) in the liver [16]. PRMT1 catalyzes the formation of NMMA and ADMA, while PRMT2 catalyzes the formation of SDMA [17]. After protein proteolysis, they are then released into the blood, where ADMA and NMMA act as competitive inhibitors of nitric oxide (NO) synthase isoforms, leading to endothelial dysfunction, which is defined as a reduction in the bioavailability of endothelial NO. Numerous studies have revealed a close relationship between the risk factors for CVD and endothelial dysfunction associated with NO deficiency [18]. The levels of circulating NMMA, ADMA, and SDMA have been extensively associated with CVD and related risk factors such as T2D, hypertension, and hypercholesterolemia [14,19,20,21]. In subjects who developed CVD or had a high CVD risk profile, elevated ADMA concentrations were able to affect the endothelial NO synthase activity [18]. Endothelial dysfunction connected with elevated ADMA concentrations has also been found in prediabetic states, such as obesity and insulin resistance, and is considered to be one of the first steps in the development of atherosclerosis [18]. In line with our data, the plasma concentrations of NMMA, ADMA, and SDMA have been shown to be increased in obese patients [22] and have been suggested to contribute to the reduced NO bioavailability reported in this population [23]. In the present study, we confirmed a strong association between circulating ADMA concentrations and BMI [24], underlying its probable role in obesity-related comorbidities [14,25,26,27]. Bariatric surgery is currently the most effective treatment for morbidly obese patients, as it produces long-term, durable weight loss and improves most obesity-related comorbidities [28,29,30,31,32]. Our results, showing that the ADMA concentration was reduced while the SDMA concentrations did not change significantly after SG or GBP, were similar to those reported previously [33]. Since SDMA originates from the PRMT2 isoform [14], we speculate that obesity affects PRMT activity and that bariatric surgery primarily reverses this activity (i.e., NMMA and ADMA production). Indeed, a recent report has demonstrated that PRMT1 plays a regulatory role in the thermogenic fat function and could represent a new therapeutic strategy against human obesity and comorbidities [34]. However, we were not able to determine the activity of PRMTs, nor that of other enzymes involved in methylarginine metabolism, since they are poorly or not secreted into the blood.

As expected, significant correlations were also found between the eGFR and concentrations of NMMA, SDMA, and DMGV [14]. Whereas SDMA is primarily excreted through the kidney, and therefore strongly associated with renal function, NMMA and ADMA are further metabolized by dimethylarginine dimethylaminohydrolase (DDAH) into CIT. In addition, ADMA can be converted to DMGV by alanine-glyoxylate aminotransferase 2 (AGXT2) [14,16]. To a lesser extent, SDMA is also a substrate for AGXT2, which catalyzes the formation of symmetric DMGV, which is excreted through the urine [35]. We did not detect symmetric DMGV, suggesting that its concentration was either below our limit of detection or that both symmetric and asymmetric DMGV were coeluted. However, our LC-MS/MS assay led to methylarginine determinations in agreement with previous reports [36,37,38]. Although the healthy individuals were enrolled a posteriori in the present study, we confirmed that the obese patients displayed elevated serum DMGV concentrations compared to the healthy individuals, an effect particularly pronounced in obese patients with T2D. Unlike ADMA, we did not observe any significant reduction in the serum DMGV level after bariatric surgery, even in patients with T2D or advanced liver fibrosis [7]. An explanation for this could be that β-aminoisobutyric acid (BAIBA), a myokine metabolite that is inversely associated with cardiometabolic disease risk [39,40], also competes with ADMA as an AGXT2 substrate. Consequently, increased concentrations of BAIBA would reduce the conversion of ADMA to DMGV [40]. Several mouse studies have shown that BAIBA supplementation sharply improves the obesity-related metabolic profile and protects against diet-induced obesity [41,42,43]. Compared to adolescents with a normal weight, the concentration of BAIBA was 29% lower in obese adolescents and was negatively correlated with insulin sensitivity [39]. However, the BAIBA concentration was not determined in the present study. Whereas DMGV is exclusively produced by AGXT2, CIT originates from DDAH1 activity, the urea cycle, and NO synthases [44]. Despite the limited design of our study, we confirmed that the plasma concentration of CIT was not increased in the obese individuals compared to the healthy individuals [45], which is in line with previous reports showing that the CIT concentration is primarily modulated by insulin resistance and diabetes but not by obesity [45,46].

The concentration of circulating DMGV is primarily associated with NAFLD and nonalcoholic steatohepatitis, but also with new-onset diabetes, T2D, and related cardiovascular complications [7,47,48]. Interestingly, DMGV has been proposed primarily as a circulating biomarker of a fatty liver, and its potential connection with T2D development seems to be more related to the underlying dysmetabolic profile, in line with our subanalysis in obese patients with T2D [47]. Since surgical weight loss significantly improves liver morphology [49], patients with NAFLD can expect significant decreases in liver volume and hepatic steatosis [50], in agreement with our clinical and anthropometric data. However, the fatty liver status of our patients was evaluated by the NAFLD fibrosis score (and confirmed by the fibrosis-4 score, data not shown), which allowed us to assess advanced fibrosis risk. A traditional computed tomography scan has been demonstrated to be able to detect moderate-to-advanced steatosis, but it has a limited diagnostic performance for fibrosis [51]. None of the target metabolites were correlated with NAFLD FS, unlike previous studies [7]. However, the number of subjects with NAFLD was too small in the present study to clearly conclude the relationship between methylarginines and a fatty liver. Interestingly, it has also been reported that NAFLD reduces the activity of DDAH in humans (the Framingham Heart Study Third Generation Cohort), leading to reduced concentrations of circulating CIT and increased concentrations of circulating ADMA; therefore, there is more available ADMA for conversion into DMGV by the enzyme AGXT2 [40]. This has been confirmed in DDAH1−/− mice that developed more severe hepatic steatosis and worse insulin resistance compared with wild-type mice [52]. Altogether, these data support the idea that the majority of our patients should not present severe liver damage, as we did not observe any decrease in the serum concentration of CIT, compared to that of the healthy individuals.

Despite improvements in both the anthropometric and the biochemical parameters, our patients remained obese after surgery. Like most biochemical parameters, the serum concentration of ARG reached that of the healthy individuals after surgery. In contrast, the serum concentrations of NMMA and ADMA were still elevated after surgery, according to the BMI. Several studies also have suggested that the investigation of plasma methylarginines and their modulations in response to different dietary macronutrients and/or chronic exercise [40] needs to be explored in greater depth. As mentioned previously, such comparisons between two different patient populations, recruited separately, should be considered with caution in our study. Further studies comparing the associations of methylarginine levels with the weight loss induced by different approaches (diet, exercise, surgery) are clearly necessary to make a ruling on this point.

Some limitations and caution in the interpretation of the results should be acknowledged in the present retrospective study. First, this is an observational study, therefore these findings should be regarded as hypothesis-generating. In addition, the group of healthy individuals was added a posteriori and was not subjected to a 12-month follow-up. Due to this study design, all samples have not been assayed after a similar storage duration, which could bias the findings. Second, the number of subjects with NAFLD was also too small to clearly conclude the relationship between DMGV and liver fat. Third, the total energy intakes of the obese patients were greatly reduced after surgery, and these drastic modifications of diet following surgical intervention may have affected various circulating biomarker levels, which represents a limitation of the current targeted metabolomic study. Similarly, the specific medications and comorbidities of the patients at baseline could represent another bias for such a metabolic study. Finally, the evaluation of NAFLD has been estimated by the NAFLD fibrosis score but not with a traditional CT scan.

## 4. Materials and Methods

### 4.1. Subjects

**Figure 5:**
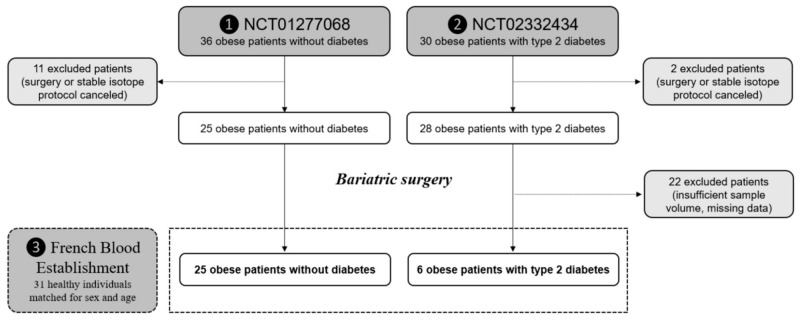
Participant flowchart.

A total of 31 obese patients referred to the Nutrition Department with an indication for bariatric surgery were used in this study (Figure 5). All of the subjects met the indication criteria for bariatric surgery [53]: (1) a BMI of ≥40 kg/m^2^ or ≥35 kg/m^2^ associated with at least one comorbidity that can be improved by surgery (arterial hypertension, obstructive sleep apnea syndrome and other severe respiratory disorders, severe metabolic disorders like T2D, disabling osteo-articular diseases, or non-alcoholic steatohepatitis); (2) the failure of a well-conducted medical, nutritional, dietetic and psychotherapeutic treatment for 6–12 months; and (3) the absence of sufficient weight loss or the absence of maintained weight loss. After a full explanation of the risks and possible benefits of each surgical procedure (the need for long-term medical and surgical follow-up, acceptable operational risk), bariatric surgery was performed by a single surgeon in the General and Endocrine Surgery Department: 16 patients undergoing GBP and 15 patients undergoing SG were consecutively included. The diet and energy intakes of each included patient were assessed 1 month before surgery and at 6 and 12 months after surgery by a dietician, using a self-administered food diary completed over three days. The clinical evaluation and laboratory tests were conducted at our Nutrition Department. The subjects were examined and followed up with iterative laboratory tests 1 month before and 6 and 12 months after surgery. Most of these obese patients were on specific medications, including oral antidiabetic agents or insulin (*n* = 7, 23%), lipid-lowering drugs (*n* = 4, 13%, statins), antihypertensive drugs (*n* = 8, 26%), and/or thyroid hormones (*n* = 8, 26%). Lipid-lowering drugs were stopped 4 weeks prior to sample collection. Patients with T2D (*n* = 9) met the diagnostic criteria for diabetes [54]. To bring some additional insights, blood samples from 31 healthy, fasting individuals, consecutively recruited in 2018 from the general population for sex- and age-matching with the obese patients at baseline (±2 years), were obtained from the Etablissement Français du Sang (French Blood Establishment, Nantes, France) (Figure 5). The healthy individuals did not display chronic diseases such as diabetes, metabolic syndrome, or cancer and were not under treatment. However, no indication of their diet was provided. This study was conducted in accordance with the Declaration of Helsinki, approved by the Research Ethics Board of Aix-Marseille University, and all subjects gave written informed consent (study numbers: NCT01277068 [2010–2014] and NCT02332434 [2015–2018]). Of note, the NCT01277068 study initially planned to investigate a third bariatric surgery (adjustable gastric banding). Given the small number of patients in this group (*n* = 6), it was decided to carry out the project only on the SG and GPB groups. Furthermore, only patients for whom blood samples were available at 6 and 12 months after surgery were studied in the current study. Blood samples were withdrawn after an overnight fast in dry tubes. After separation by traditional centrifugation (30 min, 4 °C, 3000*× g*), all serum samples were stored at −80 °C until use.

### 4.2. Chemical and Reagents

ARG, CIT, NMMA, SDMA, ADMA, and acetyl chloride were purchased from Sigma Aldrich (Saint-Quentin Fallavier, France). ^13^C-ARG, ^2^H_7_-ADMA, and ^2^H_7_-CIT were purchased from Cambridge Isotope Laboratories (Tewksbury, MA, USA). UPLC/MS-grade methanol, water, formic acid, hydrochloric acid solution (HCl, 1 M), and butanol were purchased from Biosolve (Valkenswaard, The Netherlands). DMGV was not available commercially and was synthesized in our laboratory.

### 4.3. Chemical Synthesis of Dimethylguanidino Valerate

**Figure 6:**
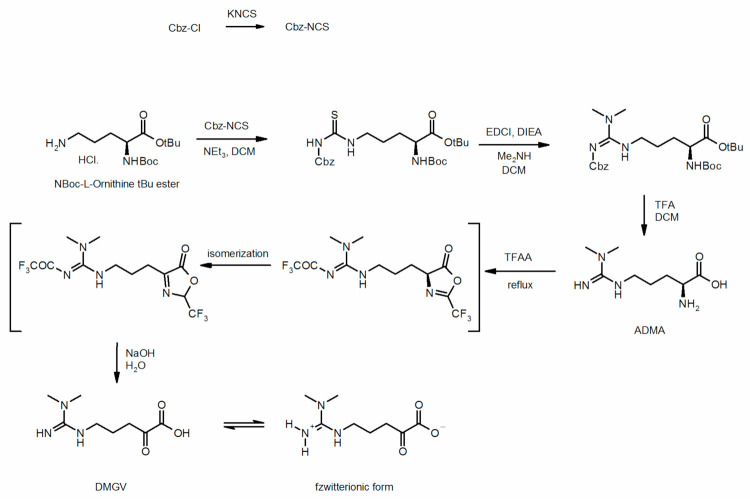
Chemical synthesis of dimethylguanidino valerate (DMGV).

DMGV synthesis began with the condensation of commercially available Nα-Boc-L-ornithine tert-butyl ester hydrochloride (Chem-Impex International, Wood Dale, IL, USA) and freshly made benzyloxycarbonylisothiocyanate (Cbz-NCS) [55] to give the protected thiourea (Figure 6). The guanidine moiety was then synthesized by condensing the thiourea functional group and dimethylamine with 1-ethyl-3-(3-dimethylaminopropyl)carbodiimide (EDCI, Sigma Aldrich, Saint-Quentin Fallavier, France) via the desulfurization and formation of a carbodiimide [56]. The full deprotection of the tert-butyl ester, tert-butyloxycarbonyl, and benzyloxycarbonyl protecting groups was accomplished by trifluoroacetic acid (TFA, Sigma Aldrich) in dichloromethane to produce ADMA exclusively. ADMA was ultimately transformed into DMGV using a modified procedure described by Klein et al. [57]: reflux in TFA anhydride followed by alkaline hydrolysis. Of note, the compound DMGV exists in a zwitterionic form in solution as well as in different cyclic hemi-aminal forms. The purity (>95%) and integrity of the synthesized DMGV were assessed by high-resolution mass spectrometry, ^1^H-nuclear magnetic resonance spectroscopy, and HPLC-UV.

### 4.4. Biochemical Measurements

Anthropometric parameters including sex, age, and BMI were recorded at each visit. TC, LDL-C, HDL-C, TG, ASAT, ALAT, platelets, albumin, glucose, and insulin were assayed in the blood samples by traditional laboratory tests. The HOMA-IR was deduced for nondiabetic patients with the following formula: fasting insulin (mIU/L) × fasting glucose (mmol/L) ÷ 22.5. The HOMA-IR was not calculated for patients with T2D to avoid miscalculation due to the antidiabetic drugs taken by the patients. Insulin resistance was established in patients for whom the HOMA-IR was >3. The NAFLD FS was calculated for the obese patients before and after surgery as follows [58]:

NAFLD FS = −1675 + (0.037 × age [*y*]) + (0.094 × BMI [kg/m^2^] + (1.13 × T2D score [yes = 1; no = 0]) + (0.99 × (ASAT/ALAT)) − (0.013 × platelets [g/L]) − (0.066 × albumin [g/L]).

A NAFLD FS > 0.676 determined the presence of advanced liver fibrosis, while a NAFLD FS < −1.455 excluded advanced liver fibrosis. A NAFLD FS between −1.455 and 0.676 was considered indeterminate. The eGFR was calculated using the Chronic Kidney Disease Epidemiology Collaboration formula [59].

### 4.5. Quantification of Methylarginine Metabolites

Analyses were performed by liquid chromatography-tandem mass spectrometry (LC-MS/MS) on a Xevo^®^ Triple-Quadrupole mass spectrometer with an electrospray ionization interface equipped with an Acquity H-Class^®^ UPLC^TM^ device (Waters Corporation, Milford, MA, USA). Methylarginine metabolites were quantified in human serum samples by a validated LC-MS/MS method [13], slightly modified. Individual stock solutions (10 mmol/L) of CIT, ARG, NMMA, ADMA, SDMA, DMGV, ^13^C-ARG, ^2^H_7_-ADMA, and ^2^H_7_-CIT were prepared in 0.1 M of HCl. A pool of unlabeled standard solutions was prepared and serially diluted in water to obtain seven standard solutions with the following ranges: 5–500 µmol/L (ARG), 0.5–50 µmol/L (CIT), and 0.02–2 µmol/L (NMMA, SDMA, ADMA, and DMGV). A solution of labeled internal standards (IS solution) consisting of ^13^C-ARG (250 µmol/L), ^2^H_7_-ADMA (5 µmol/L), and ^2^H_7_-CIT (50 µmol/L) was prepared in water. The standard solutions and serum samples (20 µL) were then extracted with 100 µL of methanol and 50 µL of the IS solution. The samples were mixed and centrifuged at 10,000× *g* at 10 °C for 15 min to remove the precipitated proteins. The supernatants were collected and dried under a gentle stream of nitrogen (45 °C). The derivatization step was performed by dissolving the dried extract in 100 µL of a freshly prepared butanol solution, containing 5% acetyl chloride and kept at 60 °C for 30 min. The solvent was then removed under a gentle stream of nitrogen (60 °C). The dried samples were dissolved in 100 µL of water containing 0.1% formic acid and injected into the LC-MS/MS system. Samples (10 µL) were injected onto an Acquity BEH-C18 column (1.7 µm; 2.1 × 100 mm, Waters Corporation) held at 60 °C, and the compounds were separated with a linear gradient of mobile phase B (0.1% formic acid in methanol) in mobile phase A (0.1% formic acid in water) at a flow rate of 400 µL/min. Mobile phase B was kept constant at 1% for 0.5 min, linearly increased from 1% to 95% for 4.5 min, kept constant for 1 min, returned to the initial condition over 0.5 min, and kept constant for 1.5 min before the next injection. The target compounds were then detected by the mass spectrometer with the electrospray interface operating in the positive ion mode (capillary voltage, 3 kV; desolvation gas (N_2_) flow, 650 L/h; desolvation gas temperature, 350 °C; source temperature, 120 °C). The multiple reaction monitoring mode was applied for MS/MS detection, as detailed in Table 4. Chromatographic peak area ratios between the unlabeled compounds and their respective IS constituted the detector responses. Standard solutions were used to plot the calibration curves for quantification. The assay linearity was expressed by the mean R^2^, which was >0.994 for all compounds (linear regression, 1/x weighting, origin excluded). The intra- and inter-assay analytical method imprecisions were assessed in spiked samples with known concentrations (three experiments, five replicates per experiment for four spiked concentrations), and were <8.4% for all compounds. Recoveries were assessed with a labeled IS and were >91%. All samples were assayed simultaneously to minimize inter-assay imprecisions. Besides, the assay has been validated for analyte stabilities after long-term storage (2 years at −80 °C) and 3 freeze/thaw cycles with recoveries greater than 91.8% for all methylarginines.

### 4.6. Statistical Analyses

Statistical analyses were performed with GraphPad Prism software (GraphPad Software Inc., La Jolla, CA, USA). Values are expressed as means ± standard deviations (SD) if normally distributed (D’Agostino–Pearson test); otherwise, they are expressed as medians (range). Unpaired data (i.e., healthy vs. obese individuals) were compared with the parametric unpaired t-test or the nonparametric Mann–Whitney test. Paired data (i.e., before/after bariatric surgery) were compared with the parametric paired *t*-test or the nonparametric Wilcoxon matched-pairs signed-rank test. Paired data from multiple groups (i.e., baseline vs. 6 months vs. 12 months) were compared with the parametric one-way analysis of variance (ANOVA) test with the Greenhouse–Geisser correction or the nonparametric Friedman test. The results were considered to be significant at *p* < 0.05. Due to the multiple testing, p-values have been corrected for false discovery rate (Benjamini–Hochberg correction) [60], and the sample size was considered too small to perform additional adjustments for covariates, as previously recommended [61]. The Spearman’s rank correlation coefficient was computed with the R software and displayed with the “corrplot package” (https://cran.r-project.org/web/packages/corrplot/corrplot.pdf, accessed on 30 October 2021). 

## 5. Conclusions

Methylarginines are promising biomarkers of metabolic disorders. Most retrospective and prospective population-based studies have focused primarily on ADMA and SDMA, and only more recently on DMGV. Here, we examined a set of methylarginines (ARG, CIT, NMMA, SDMA, and ADMA) in a small cohort of obese patients with various phenotypes and who underwent bariatric surgery. Our data confirmed that obesity dramatically increased the concentrations of circulating methylarginines in parallel with the traditional markers of dyslipidemia, insulin resistance, and a fatty liver. Bariatric surgery markedly improved the dysmetabolic profiles and sharply reduced the concentrations of ADMA and its related precursor metabolites (ARG and NMMA), which led to reduced atherogenic profiles in the obese patients. No significant difference was found for DMGV, CIT, or SDMA after surgery. This suggests that methylarginines follow different metabolic patterns, which could be helpful for the better stratification of cardiometabolic disorders in obese patients. Hence, further studies in larger cohorts, targeting a full set of methylarginines, are warranted to better understand this metabolic pathway and the metabolic disorders underlying a pathology as complex as obesity.

## Figures and Tables

**Table 1:** Clinical characteristics of the subjects at baseline.

	Healthy Controls	Obese Patients at Baseline	*p*-Value ^1^
*n*	31	31	-
Male/Female	9/22	9/22	0.713
Age, years	40 ± 10	41 ± 9	0.652
BMI, kg/m^2^	22 ± 3	45 ± 6	0.003
TC, mg/dL	187 ± 31	189 ± 31	0.888
TG, mg/dL	81 [45–176]	108 [59–625]	0.003
HDL-C, mg/dL	73 [50–90]	0.003	
LDL-C, mg/dL	95 ± 34	120 ± 21	0.003
Glucose, mg/dL	77 [58–103]	93 [72–341]	0.007
Insulin, mIU/L	15 [3–48]	19 [6–55]	0.031
HOMA-IR	2.9 [0.7–12.1]	4.9 [2.9–23.9]	0.006
ASAT, IU/L	14 [3–44]	19 [11–64]	0.027
ALAT, IU/L	22 [13–98]	28 [5–114]	0.005
eGFR, mL/min/1.73 m^2^	Not available	99 ± 19	-
NAFLD FS	Not available	−0.662 ± 1.259	-

Values are expressed as the means ± SD if they are normally distributed, otherwise expressed as medians (range). *n*, numbers of subjects; BMI, body mass index; TC, total cholesterol; TG, triglycerides; HDL-C, high-density lipoprotein cholesterol; LDL-C, low-density lipoprotein cholesterol; ASAT, aspartate aminotransferase; ALAT, alanine aminotransferase; HOMA-IR, homeostasis model assessment of insulin resistance; eGFR, estimated glomerular filtration rate; NAFLD FS, nonalcoholic fatty liver disease fibrosis score. ^1^ For a normal distribution of values, the unpaired t-test was used; otherwise, the Mann–Whitney test was used. *p*-values have been corrected for false discovery rate (Benjamini–Hochberg correction).

**Table 2:** Effect of bariatric surgery on the clinical parameters of the patients in comparison to the baseline values.

	6 Months		12 Months	
Parameter	% of Change	*p*-Value ^1^	% of Change	*p*-Value ^1^
BMI	−23.8	0.002	−28.7	0.002
TC	−9.6	0.002	−11.4	0.002
TG	−26.4	0.004	−36.2	0.004
HDL-C	+10.6	0.999	+31.1	0.027
LDL-C	−11.7	0.038	−18.9	0.008
Glucose	−17.9	0.036	−21.8	0.011
Insulin	−55.4	0.002	−63.7	0.003
HOMA-IR	−62.0	0.002	−71.4	0.003
ASAT	−16.4	0.043	−15.3	0.031
ALAT	−30.8	0.009	−30.9	0.004
eGFR	+11.1	0.002	+5.9	0.004
NAFLD FS	−66.7	0.011	−92.4	0.002

Values are expressed as mean changes (*n* = 31). BMI, body mass index; TC, total cholesterol; TG, triglycerides; HDL-C, high-density lipoprotein cholesterol; LDL-C, low-density lipoprotein cholesterol; ASAT, aspartate aminotransferase; ALAT, alanine aminotransferase; HOMA-IR, homeostasis model assessment of insulin resistance; eGFR, estimated glomerular filtration rate; NAFLD FS, nonalcoholic fatty liver disease fibrosis score. ^1^ For a normal distribution of values, the one-way ANOVA test with the Greenhouse–Geisser correction was used; otherwise, the Friedman test was used. *p*-values have been corrected for false discovery rate (Benjamini–Hochberg correction).

**Table 3:** Comparison of the clinical characteristics and biomarker concentrations between the healthy controls and the obese patients after bariatric surgery (12 months post-surgery).

Parameter	Healthy Controls	Obese Patients (12 Months Post-Surgery)	*p*-Value ^1^
*n*	31	31	-
Male/Female	9/22	9/22	0.785
Age, years	40 ± 10	41 ± 9	0.698
BMI, kg/m^2^	22 ± 3	32 ± 5	0.003
TC, mg/dL	187 ± 31	167 ± 30	0.020
TG, mg/dL	81 [45–176]	68 [36–125]	0.020
HDL-C, mg/dL	73 [50–121]	54 [37–79]	0.003
LDL-C, mg/dL	95 ± 34	97 ± 24	0.815
Glucose, mg/dL	77 [58–103]	83 [70–106]	0.089
Insulin, mIU/L	15 [3–48]	8 [2–14]	0.003
HOMA-IR	2.9 [0.7–12.1]	1.7 [0.4–2.9]	0.038
ASAT, IU/L	14 [3–44]	16 [11–37]	0.831
ALAT, IU/L	22 [13–98]		0.020
eGFR, mL/min/1.73 m^2^	Not available		-
ARG, µmol/L	157 ± 24	16 [5–36]	0.972
NMMA, nmol/L	466 ± 118	105 ± 16	0.003
CIT, µmol/L	33 ± 5	37 ± 9	0.089
ADMA, nmol/L	820 ± 176	1066 ± 202	0.003
SDMA, nmol/L	685 ± 103	800 ± 156	0.003
DMGV, nmol/L	324 [87–891]	483 [245–1251]	0.003

Values are expressed as means ± SD if they are normally distributed; otherwise, they are expressed as medians (range). *n*, number of subjects; BMI, body mass index; TC, total cholesterol; TG, triglycerides; HDL-C, high-density lipoprotein cholesterol; LDL-C, low-density lipoprotein cholesterol; ASAT, aspartate aminotransferase; ALAT, alanine aminotransferase; HOMA-IR, homeostasis model assessment of insulin resistance; eGFR, estimated glomerular filtration rate; NAFLD FS, nonalcoholic fatty liver disease fibrosis score; ARG, arginine; CIT, citrulline; NMMA, N-monomethylarginine; ADMA, asymmetric dimethylarginine; SDMA, symmetric dimethylarginine; DMGV, dimethylguanidino valerate. ^1^ For a normal distribution of values, the unpaired t-test was used; otherwise; the Mann–Whitney test was used. *p*-values have been corrected for false discovery rate (Benjamini–Hochberg correction).

**Table 4:** Multiple reaction monitoring (MRM) transitions used for LC-MS/MS detection.

Compound	MRM Transition (*m*/*z*)	Cone/Collision (V)
ARG	231.1 → 69.9	30/20
^13^C-ARG (IS)	232.1 → 69.9	30/20
CIT	232.1 → 112.9	30/20
^2^H_7_-CIT (IS)	239.1 → 119.9	30/20
NMMA	245.1 → 69.9	30/20
ADMA	259.1 → 214.1	30/20
SDMA	259.1 → 228.1	30/20
DMGV	258.2 → 113.9	30/25
^2^H_7_-ADMA (IS)	266.2 → 221.1	30/20

ARG, arginine; IS, internal standard; CIT, citrulline; NMMA, N-monomethylarginine; ADMA, asymmetric dimethylarginine; SDMA, symmetric dimethylarginine; DMGV, dimethylguanidino valerate.

## Data Availability

The data presented in this study are available upon reasonable request to the corresponding author. The data are not publicly available due to the ethical confidentiality related to such clinical studies.
